# Soluble vascular endothelial cadherin: a promising marker of critical illness?

**DOI:** 10.1186/s13054-019-2343-7

**Published:** 2019-02-19

**Authors:** Ruyuan Zhang, Ranran Li, Yaoqing Tang

**Affiliations:** Department of Critical Care Medicine, Rui Jin Hospital, Shanghai Jiao Tong University School of Medicine, Shanghai, 200025 China

Endothelial cells play a major role in the pathophysiology of sepsis. The recently published study by Yu et al. [1] demonstrated that soluble vascular endothelial cadherin (VE-cadherin) is associated with severe acute kidney injury and with more severe organ dysfunction in patients with sepsis. This study, together with ours [2] and others’ studies cited in the article by Yu et al. [1], suggests that soluble VE-cadherin may function as a promising marker of critical illness. VE-cadherin is a major component of adherens junctions between endothelial cell-cell contact, mediating Ca^2+^-dependent, homophilic endothelial cell-cell adhesion, which plays a critical role in the maintenance and regulation of the endothelial barrier. VE-cadherin can function as three main forms: endocytosis of full VE-cadherin, shedding of extracellular domain (ectodomain shedding), or redistribution within cell membrane. All these three forms of VE-cadherin regulation can result in disruption of VE-cadherin-mediated endothelial cell-cell adhesion and endothelial barrier. Since it is unlikely to evaluate full VE-cadherin endocytosis or redistribution within cell membrane in clinical situations, soluble VE-cadherin in circulation after VE-cadherin ectodomain shedding may function as a marker of breakdown of endothelial adherens junctions in patients, which results in capillary leakage, tissue edema, and organ dysfunction.

Studies in the literature show that inflammatory mediators and growth factors, such as LPS, TNFα, and VEGF, can induce several forms of VE-cadherin changes simultaneously. In addition to its adhesive properties, VE-cadherin acts by transferring intracellular signals, which may regulate the release or activation of matrix metalloproteinases leading to cleavage of VE-cadherin ectodomain [3]. Alternatively, the outside-in signaling of VE-cadherin may activate the endocytic machinery resulting in the internalization of VE-cadherin. Using the approach of limiting the activation of other confounding signaling pathways known to be initiated by inflammatory mediators or growth factors, disruption of VE-cadherin-mediated adhesion by a small inhibitory peptide, which binds to VE-cadherin extracellular 1 module and disrupts VE-cadherin trans-interaction, induced the activation of Src [4], a non-receptor protein tyrosine kinase known to induce VE-cadherin phosphorylation and endocytosis contributing to a dynamic state of adherens junctions. These observations raise a possibility, although not directly addressed before, that there may be a possible interplay between these forms of VE-cadherin changes, making it more attractive to suppose that plasma soluble VE-cadherin can reflect the profound disruption of endothelial barrier in vivo. However, it is interesting to note that plasma levels of soluble VE-cadherin are not associated with fluid accumulation in the present study. Currently, the reason for this is not clear. Septic animal experiments showed a dynamic and organ-specific change of VE-cadherin mRNA and protein. In addition, several clinical questions remain. Can we differentiate between infectious and noninfectious causes of organ dysfunction using soluble VE-cadherin? Is there any difference on the intensity of endothelial activation and damage, as judged by shedding of VE-cadherin, in septic patients who have microbiologically confirmed infection compared with patients with culture-negative sepsis? Thus, while soluble VE-cadherin holds a promise as a marker of critical illness, further investigations are still needed to confirm and extend the existing findings.
